# Proteomics in Pancreatic Cancer Research

**DOI:** 10.1155/2011/365350

**Published:** 2011-08-14

**Authors:** Ruihui Geng, Zhaoshen Li, Shude Li, Jun Gao

**Affiliations:** Department of Gastroenterology, Changhai Hospital, The Second Military Medical University, Shanghai 200433, China

## Abstract

Pancreatic cancer is a highly aggressive malignancy with a poor prognosis and deeply affects the life of people. Therefore, the earlier diagnosis and better treatments are urgently needed. In recent years, the proteomic technologies are well established and growing rapidly and have been widely applied in clinical applications, especially in pancreatic cancer research. In this paper, we attempt to discuss the development of current proteomic technologies and the application of proteomics to the field of pancreatic cancer research. This will explore the potential perspective in revealing pathogenesis, making the diagnosis earlier and treatment.

## 1. Introduction

Pancreatic cancer is a highly aggressive malignancy with a poor prognosis; however, the present treatments are incapable of producing a desired effect. The patients generally die within six months after diagnosis, and the overall five-year survival rate is less than 5% [1]. Its incidence is increasing in China and other countries. Therefore, the earlier diagnosis and better treatments are urgently needed. In recent years, the development of quantitative proteomics technology has stimulated considerable interest in applying the technology for clinical applications, such as revealing pathogenesis, making the diagnosis earlier, and treatment. In this paper, we provide an overview of recent findings in proteomics of pancreatic cancer.

## 2. The Outline of Proteomics Research

The term “proteome” was first used in 1994 and describes the entire set of proteins expressed by a given genome, cell, tissue, or organism [2]. Initially, the word proteomics referred to the techniques used to analyze a large number of proteins at the same time; however, at present this word covers any approach that yields information on the abundance, properties, interactions, activities, or structures of proteins in a sample.

Proteomics is the main tool for proteome research. The rapid development of proteomics was made possible by the progress in analytical instrumentation, especially in mass spectrometry, and it is increasingly becoming the foundation in leading scientific workgroups and in clinical research labs.

Current proteomics research can be defined as two types [3] (i) cell-mapping proteomics which aims to define protein-protein interactions to build a picture of the complex networks that constitute intracellular signaling pathways and (ii) protein expression proteomics and which monitors global expression of large numbers of proteins within a cell type or tissue and quantitatively identifies how patterns of expression change in different circumstances.

## 3. The Methods of Proteomics Analysis

A proteomics analysis usually consists of two steps, protein separation and protein identification. Several technologies, such as two-dimensional polyacrylamide gel electrophoresis (2DE) and other nongel-based separation techniques, mass spectrometry (MS), and protein microarrays, are relatively common. Moreover, phosphoproteomics is a novel method which makes fully use of these technologies and is frequently used in medical studies, such as signal transduction and the studies of cancer. It is an important complement of the classic methods to study multiple kinases and its products. 

### 3.1. Gel-Based Separation Techniques

Gel-based methods are well-defined techniques in the proteomic field and are the most commonly used. The central method for proteomic analysis is two-dimensional gel electrophoresis (2DE). The technique was established in 1975 and is still an important research tool. It is developed to separate complex protein mixtures into orthogonal separated components by isoelectric point and molecular weight [4]. 

Two-dimensional difference gel electrophoresis (2D-DIGE) is a differential method for comparing two protein samples. It combines conventional 2DE with the sensitivity of fluorescent protein labeling for analytical gels and mass finger print analysis by mass spectrometry for preparative gels used for protein identification. Rong et al. [5] used affinity column enrichment and DIGE to identify proteins differentially expressed in serum from pancreatic cancer patients. They found that mannose-binding lectin 2 and myosin light chain kinase 2 protein were overexpressed in serum from pancreatic cancer patients, and these proteins might be potential biomarkers of pancreatic cancer. 2D-DIGE effectively solves the reproducibility setback of 2-DE, giving more accurate and reliable quantification information of protein abundance. An additional advantage of DIGE is that it can detect isoform changes, such as posttranslational modification or alternative spicing [6].

### 3.2. Nongel-Based Separation Techniques

Non-gel-based separation techniques provide additional information which is used to detect low-abundant or hydrophobic (membrane) proteins. Alternative approaches use gel-free techniques by combining liquid chromatography and mass spectrometry. The significant advantages of these techniques over 2DE are potential high-throughput capabilities, possibility of full automation, direct integration with MS, higher sensitivity, and the smaller amount of starting material needed [7]. 

Liquid chromatographic (LC) methods are most used to fractionate samples, which is based on two or more biophysical characteristics, such as surface charge, hydrophobicity, or affinity to particular compounds. Furthermore, 2D chromatographic strategy termed multidimensional protein identification technology (MudPIT) has been extensively applied to proteomics analyses at a peptide level. Surface-enhanced laser desorption/ionization time-of-flight mass spectrometry (SELDI-TOF MS), normal-phase/reversed-phase high-performance liquid chromatography (NP/RP-HPLC), and combined fractional diagonal chromatography (COFRADIC) are usually applied in medical research. It should be emphasized that none of these methods enables conclusions to be drawn regarding relative protein concentrations [8].

MS is an analytical technique that measures the mass-to-charge ratio (m/z) of charged particles. It is used to determine masses of particles, elemental composition of a sample or molecule, and the chemical structures of molecules, such as peptides and other chemical compounds. MS plays a central role in proteomics and is emerging as the preferred method for the characterization of the protein components [9]. There are many types of mass spectrometers that can be used for proteomic studies, such as time-of-flight (TOF), quadrupole (Q), triple quadrupole or linear ion trap (LIT), ion trap (IT), Fourier transform ion cyclotron resonance (FTICR), and Orbitrap. Being highly sensitive and extremely accurate, MS is used to discover early biomarkers of cancer. The limitations of MS are low-throughput capabilities, large protein samples and insufficiency of low-abundance proteins sensibility. Moreover, the type of MS technique used can affect the interpretation of the data retrieved which might lead to an element of subjectivity [10]. 

The development of nongel-based “shotgun” proteomic techniques was the remarkable advances in proteomic technologies in the last decade. MudPIT has provided powerful tool to study large-scale protein expression and characterization in complex biological systems [11, 12]. Current methods for protein quantification mostly involve the use of electrospray ionization (ESI), matrix-assisted laser desorption ionization (MALDI ), SELDI, isotope-coded affinity tags (ICAT and iTRAQ), isotope-coded protein labeling (ICPL), tandem mass tags (TMT) and ^15^N/^14^N metabolic labeling, and so forth. These methods provide valuable flexibility to study protein changes in complex samples, and can measure the slight changes (<2-fold) between samples [13]. The promising prospects of the quantitative proteomics are gaining more and more interest in biomedical research. Particularly, precise quantitative measurements are key to understand the relationships between normal cellular biology and the aberrant biology observed in cancer. Recently, iTRAQ has been optimized to quantificate proteins in pancreatic cancer serum [14]. Zhao et al. [15] used stable isotope labeling technology for quantitative study to measure protein synthesis in pancreatic cancer cells. However, most quantification approaches are far from perfect, such as increased time and complexity of sample preparation, requirement for higher sample concentration, and high cost of the reagents.

### 3.3. Protein Microarrays

Protein microarray provides a multiplex approach to identify protein-protein interactions, the substrates of protein kinases, transcription factor protein activation, or the targets of biologically active small molecules. Three types of protein microarrays currently used to study the biochemical activities of proteins are analytical microarrays, functional microarrays, and reverse phase microarrays [16]. The most common protein microarray is the antibody microarray. Related microarray technologies also include DNA microarrays, cellular microarrays, antibody microarrays, tissue microarrays, and chemical compound microarrays [17]. Protein microarray is increasingly applied for high-throughput protein analyses in many research areas. Schröder et al. [18] recently used an optimized extensive protein microarray for the serum and urine samples of pancreatic cancer patients. However, the detection of low-abundance proteins yet remains a problem, and the sensitivity and reproducibility need to be improved.

### 3.4. Phosphoproteome

Phosphoproteomics is a branch of proteomics, and it is useful in characterizing proteins containing a phosphate group as a posttranslational modification. Compared to expression analysis, phosphoproteomics provides two additional layers of information. (i) It provides clues on which protein or pathway might be activated. (ii) It indicates which proteins might be potential drug targets. It can analyze the entire phosphorylation-based signaling networks [19]. For example, by phosphoproteome and transcriptome analyses, Nagashima et al. [20] recently found that histidine-rich-glycoprotein- (HRG-) stimulated molecular activation was significantly related to cancer pathways in pancreatic cancer.

Enrichment strategies and MS analysis are usually applied in phosphoproteomics. However, there are still a few limitations for these techniques. Firstly, isolation methods need to be improved, for instance, antiphosphotyrosine antibodies are incapable of distinguishing between isolating tyrosine-phosphorylated proteins and proteins associated with tyrosine-phosphorylated proteins. Secondly, some relevant proteins may be missed because the extraction conditions are not encompassing. Therefore, proteins in very low abundance, or phosphorylated as a target for rapid degradation, may be missed [21].

## 4. Proteomics Studies in Pancreatic Cancer

### 4.1. In Search of Pancreatic Cancer Pathogenesis

The transforming growth factor-beta (TGF-beta)-Smad signaling pathway has a pivotal role in inhibiting the growth of tumor cells. Ijichi et al. reported that the mutation or deletion of the Smad4 gene is found in 50% of pancreatic cancers [22]. Jazag et al. [23] established Smad4 knockdown (S4KD) pancreatic cancer cell lines and screened for the targeted molecules downstream of TGF-beta using cDNA microarray and found that the signaling pathways were different according to the Smad4 status. By 2DE and LC-MS/MS, Mikuriya et al. [24] observed that 4 of 11 spots overexpressed in pancreatic cancer were the enzymes involved in glycolytic pathway, while increased glycolysis has been regarded as the effect of intratumoral hypoxia and is possibly associated with tumor invasion, metastasis, or resistance to therapies. Dai et al. [25] studied the proteome of pancreatic cancer stem cells using a capillary scale shotgun technique. By coupling off-line capillary isoelectric focusing (cIEF) with nano-reversed phase liquid chromatography (RPLC) followed by spectral counting peptide quantification, they identified 169 differentially expressed proteins, of which 24% are upregulated. Ingenuity pathway analysis of these differential expression signatures further suggested significant involvement of signaling pathways related to apoptosis, cell proliferation, inflammation, and metastasis. It was also noteworthy that the expression of mucins-1 was upregulated, which could enhance invasiveness of pancreatic cancer cells by inducing epithelial to mesenchymal transition [26].

Diabetes mellitus is associated with pancreatic cancer in more than 80% of the cases. Basso et al. [27] analyzed a series of pancreatic cancer cell lines in conditioned media, pancreatic cancer patients' peripheral and portal sera, and compared them with controls' and chronic pancreatitis patients' sera by MALDI-TOF. A tumor-derived peptide of 14 amino acids sharing a 100% homology with an S-100 calcium-binding protein was identified, which was therefore suggested to be a pancreatic cancer-associated diabetogenic factor. This study might provide new insights into the mechanism of the pancreatic cancer-associated diabetes. It could be referred that many proteins might play certain important roles in pancreatic cancer's genesis. 

### 4.2. In Discovery of the Pancreatic Cancer Biomarkers

The applications of proteomics techniques can screen and identify the immunogenic membrane antigens in high-risk population for early diagnosis of pancreatic cancer. The serum glycosylation marker CA19-9 is the most commonly used marker in pancreatic cancer. The results of Navaglia et al. [28] suggested that the combination of CA 19-9 and SELDI-TOF/MS features could improve the diagnostic accuracy of CA 19-9. SELDI-TOF/MS allows identification of new peptides, which in addition to CA 19-9 enables the correct classification of the vast majority of patients with pancreatic cancer. By immunohistochemistry, oligonucleotide microarray, and serial analysis of gene, Koopmann et al. [29] recently also found that serum macrophage inhibitory cytokine-1 (MIC-1) is a new marker of pancreatic cancer. The combination of MIC-1 and CA19-9 significantly improved the diagnostic accuracy, with a sensitivity of 70% and a specificity of 85%. Matsubara et al. [30] identified a significant decrease of the plasma CXC chemokine ligand 7(CXCL7) level in pancreatic cancer. The combination of CA19-9 with CXCL7 improved the discriminatory power over the former alone for pancreatic cancer diagnosis. These findings may provide a new diagnostic option for pancreatic cancer and facilitate early detection of the disease. Other proteins were also identified as new potential discriminating markers of pancreatic cancer, such as phosphoglycerate kinase (PGK) 1, histone H4 cyclin I, Rab GDP dissociation inhibitor b (GDI2), and serotransferrin platelet factor 4 (PF4) [31–33].

Primarily by 2-DE analyses, pancreatic juice was extensively studied and led to discovery of several pancreatic enzymes in the late 1970s and 1980s [34]. At present, more and more techniques are applied in pancreatic juice research. Tian et al. [35] carried out DIGE and MS/MS to compare the pancreatic juice profiling from pancreatic ductal adenocarcinoma (PDAC) patients and cancer-free controls. The present proteome analysis revealed that matrix metalloproteinase-9 (MMP-9), oncogene DJ1 (DJ-1), and alpha-1B-glycoprotein precursor (A1BG) proteins were elevated in pancreatic juice from PDAC patients, which suggested their further utility in PDAC diagnosis. Kojima reported that specific urine biomarkers distinguished malignancy from chronic inflammation using the method of MALDI-MS and MS/MS [36].

### 4.3. In the Treatment of Pancreatic Cancer

The level of activation/repression of multiple regulated proteins involved in the PDAC processes correlates with the growth inhibition and the apoptotic response of the cells subjected to single or combined drug treatment [37]. Mori-Iwamoto et al. [38] performed proteomic analysis (2-DE, LC-MS/MS) and found that seven proteins, including heat stress protein (HSP) 27, peroxiredoxin 2, endoplasmic reticulum protein (Erp) 29 precursor, 6-phosphogluconolactonase, triosephosphate isomerase, alpha enolase, and nucleophosmine, could play a role in estimating the sensitivity of pancreatic cancer to gemcitabine. The sensitivity to gemcitabine was restored by knocked down HSP27 in resistant pancreatic cancer cells, while increased HSP27 expression was related to higher resistibility. Furthermore, KNK437, an HSP inhibitor, downregulated HSP27 of pancreatic cancer cells and enhanced the cytotoxic effect of gemcitabine [39]. Hereby, HSP27 may be involved in the resistance to gemcitabine and could be a possible predictor of the response to gemcitabine-based regimen. Kuramitsu et al. [40] identified the proteins mediating the poor response of pancreatic cancer to gemcitabine by 2DE and MS.

Taniuchi et al. [41] introduced immunohistochemistry, MS analysis, semiquantitative RT-PCR, and Northern blot analysis into identifying novel molecular targets for the treatment of PDAC. Their results suggested that collaboration of kinesin RAB6KIFL and discs large homologue 5 (DLG5) was likely to be involved in pancreatic carcinogenesis. Synuclein-gamma overexpression was observed in pancreatic cancer with perineural invasion and lymph node metastasis. It was found to be the only independent predictor of diminished overall survival and the strongest negative indicator of disease-free survival [42]. These molecules might be promising targets for development of new therapeutic strategies and allow a rational and individualized therapy for pancreatic cancer [43]. 

 The study of Shields et al. [44] on retinoblastoma-binding protein (RBBP) 9, a tumor-associated serine hydrolase, is worth noting. By using LC/LC MS/MS, activity-based proteomic profiling (ABPP) coupled to MudPIT, immunoprecipitation, and immunoblot, these authors found that RBBP9 displayed elevated activity in pancreatic carcinomas, which overcame TGF-beta-mediated antiproliferative signaling by reducing Smad2/3 phosphorylation. The expression of E-cadherin was required in this process, and the decrease of the levels of E-cadherin will lead to reducing the integrity of tumor cell-cell junctions. These data demonstrated that functional proteomics has a potential benefit in the identification of new therapeutic targets.

## 5. Conclusion

Proteomics is a rapidly developing science and offers assistance in the diagnosis and treatment of pancreatic cancer. However, certain potential difficulties have also been recognized. The sensitivity, specificity, and reproducibility of the available molecular markers are still below the expectation [45]. With the improvement of proteomics technology, this branch of science would surely become the potential and essential tool for the early diagnosis and treatment of pancreatic cancer.

## References

[1] Hidalgo M (2010). Pancreatic cancer. *The New England Journal of Medicine*.

[2] Wasinger VC, Cordwell SJ, Cerpa-Poljak A (1995). Progress with gene-product mapping of the mollicutes: mycoplasma genitalium. *Electrophoresis*.

[3] Moritz RL, Skandarajah AR, Ji H (2005). Proteomic analysis of colorectal cancer: prefractionation strategies using two-dimensional free-flow electrophoresis. *Comparative and Functional Genomics*.

[4] Rabilloud T (2002). Two-dimensional gel electrophoresis in proteomics: old, old fashioned, but it still climbs up the mountains. *Proteomics*.

[5] Rong Y, Jin D, Hou C (2010). Proteomics analysis of serum protein profiling in pancreatic cancer patients by DIGE: up-regulation of mannose-binding lectin 2 and myosin light chain kinase 2. *BMC Gastroenterology*.

[6] Minden J (2007). Comparative proteomics and difference gel electrophoresis. *BioTechniques*.

[7] Liumbruno GM (2008). Proteomics: applications in transfusion medicine. *Blood Transfusion*.

[8] Page MJ, Griffiths TA, Bleackley MR (2006). Proteomics: applications relevant to transfusion medicine. *Transfusion Medicine Reviews*.

[9] Cravatt BF, Simon GM, Yates JR (2007). The biological impact of mass-spectrometry-based proteomics. *Nature*.

[10] Conrad DH, Goyette J, Thomas PS (2008). Proteomics as a method for early detection of cancer: a review of proteomics, exhaled breath condensate, and lung cancer screening. *Journal of General Internal Medicine*.

[11] Motoyama A, Yates JR (2008). Multidimensional LC separations in shotgun proteomics. *Analytical Chemistry*.

[12] Domon B, Aebersold R (2006). Mass spectrometry and protein analysis. *Science*.

[13] Roe MR, Griffin TJ (2006). Gel-free mass spectrometry-based high throughput proteomics: tools for studying biological response of proteins and proteomes. *Proteomics*.

[14] Tonack S, Aspinall-O’Dea M, Jenkins RE (2009). A technically detailed and pragmatic protocol for quantitative serum proteomics using iTRAQ. *Journal of Proteomics*.

[15] Zhao Y, Lee WN, Lim S (2009). Quantitative proteomics: measuring protein synthesis using ^15^N amino acid labeling in pancreatic cancer cells. *Analytical Chemistry*.

[16] Hall DA, Ptacek J, Snyder M (2007). Protein microarray technology. *Mechanisms of Ageing and Development*.

[17] Xu Q, Lam KS (2003). Protein and chemical microarrays-powerful tools for proteomics. *Journal of Biomedicine and Biotechnology*.

[18] Schröder C, Jacob A, Tonack S (2010). Dual-color proteomic profiling of complex samples with a microarray of 810 cancer-related antibodies. *Molecular and Cellular Proteomics*.

[19] Lim YP (2005). Mining the tumor phosphoproteome for cancer markers. *Clinical Cancer Research*.

[20] Nagashima T, Oyama M, Kozuka-Hata H (2008). Phosphoproteome and transcriptome analyses of ErbB ligand-stimulated MCF-7 cells. *Cancer Genomics and Proteomics*.

[21] Johnson SA, Hunter T (2004). Phosphoproteomics finds its timing. *Nature Biotechnology*.

[22] Ijichi H, Otsuka M, Tateishi K (2004). Smad4-independent regulation of p21/WAF1 by transforming growth factor-*β*. *Oncogene*.

[23] Jazag A, Ijichi H, Kanai F (2005). Smad4 silencing in pancreatic cancer cell lines using stable RNA interference and gene expression profiles induced by transforming growth factor-*β*. *Oncogene*.

[24] Mikuriya K, Kuramitsu Y, Ryozawa S (2007). Expression of glycolytic enzymes is increased in pancreatic cancerous tissues as evidenced by proteomic profiling by two-dimensional electrophoresis and liquid chromatography-mass spectrometry/mass spectrometry. *International Journal of Oncology*.

[25] Dai L, Li C, Shedden KA (2010). Quantitative proteomic profiling studies of pancreatic cancer stem cells. *Journal of Proteome Research*.

[26] Roy LD, Sahraei M, Subramani DB (2011). MUC1 enhances invasiveness of pancreatic cancer cells by inducing epithelial to mesenchymal transition. *Oncogene*.

[27] Basso D, Greco E, Fogar P (2005). Pancreatic cancer-associated diabetes mellitus: an open field for proteomic applications. *Clinica Chimica Acta*.

[28] Navaglia F, Fogar P, Basso D (2009). Pancreatic cancer biomarkers discovery by surface-enhanced laser desorption and ionization time-of-flight mass spectrometry. *Clinical Chemistry and Laboratory Medicine*.

[29] Koopmann J, Buckhaults P, Brown DA (2004). Serum macrophage inhibitory cytokine 1 as a marker of pancreatic and other periampullary cancers. *Clinical Cancer Research*.

[30] Matsubara J, Honda K, Ono M (2011). Reduced plasma level of CXC chemokine ligand 7 in patients with pancreatic cancer. *Cancer Epidemiology Biomarkers and Prevention*.

[31] Patwa TH, Li C, Poisson LM (2009). The identification of phosphoglycerate kinase-1 and histone H4 autoantibodies in pancreatic cancer patient serum using a natural protein microarray. *Electrophoresis*.

[32] Sun ZL, Zhu Y, Wang FQ (2007). Serum proteomic-based analysis of pancreatic carcinoma for the identification of potential cancer biomarkers. *Biochimica et Biophysica Acta*.

[33] Fiedler GM, Leichtle AB, Kase J (2009). Serum peptidome profiling revealed platelet factor 4 as a potential discriminating peptide associated with pancreatic cancer. *Clinical Cancer Research*.

[34] Scheele GA (1975). Two dimensional gel analysis of soluble proteins. Characterization of guinea pig exocrine pancreatic proteins. *Journal of Biological Chemistry*.

[35] Tian M, Cui YZ, Song GH (2008). Proteomic analysis identifies MMP-9, DJ-1 and A1BG as overexpressed proteins in pancreatic juice from pancreatic ductal adenocarcinoma patients. *BMC Cancer*.

[36] Kojima K, Asmellash S, Klug CA (2008). Applying proteomic-based biomarker tools for the accurate diagnosis of pancreatic cancer. *Journal of Gastrointestinal Surgery*.

[37] Cecconi D, Donadelli M, Scarpa A (2005). Proteomic analysis of pancreatic ductal carcinoma cells after combined treatment with gemcitabine and trichostatin A. *Journal of Proteome Research*.

[38] Mori-Iwamoto S, Kuramitsu Y, Ryozawa S (2007). Proteomics finding heat shock protein 27 as a biomarker for resistance of pancreatic cancer cells to gemcitabine. *International Journal of Oncology*.

[39] Taba K, Kuramitsu Y, Ryozawa S (2011). KNK437 downregulates heat shock protein 27 of pancreatic cancer cells and enhances the cytotoxic effect of gemcitabine. *Chemotherapy*.

[40] Kuramitsu Y, Taba K, Ryozawa S (2010). Identification of up- and down-regulated proteins in gemcitabine-resistant pancreatic cancer cells using two-dimensional gel electrophoresis and mass spectrometry. *Anticancer Research*.

[41] Taniuchi K, Nakagawa H, Nakamura T (2005). Down-regulation of RAB6KIFL/KIF20A, a kinesin involved with membrane trafficking of discs large homologue 5, can attenuate growth of pancreatic cancer cell. *Cancer Research*.

[42] Hibi T, Mori T, Fukuma M (2009). Synuclein-*γ* is closely involved in perineural invasion and distant metastasis in mouse models and is a novel prognostic factor in pancreatic cancer. *Clinical Cancer Research*.

[43] Löhr JM, Faissner R, Findeisen P (2006). Proteome analysis-basis for individualized pancreatic carcinoma therapy?. *Internist*.

[44] Shields DJ, Niessen S, Murphy EA (2010). RBBP9: a tumor-associated serine hydrolase activity required for pancreatic neoplasia. *Proceedings of the National Academy of Sciences of the United States of America*.

[45] Fry LC, Mönkemüller K, Malfertheiner P (2008). Molecular markers of pancreatic cancer: development and clinical relevance. *Langenbeck’s Archives of Surgery*.

